# The relationship between place attachment and pro-environmental behavioral intentions: the mediating role of nature connectedness

**DOI:** 10.3389/fpsyg.2025.1678177

**Published:** 2026-01-12

**Authors:** Baorui Chang, Huanyu Sheng, Yanhan Wei, Jiandong Fang, Yaping Wen

**Affiliations:** 1 School of Educational Science, Guangdong Polytechnic Normal University, Guangzhou, China; 2 Department of Psychology, Faculty of Education, Guangxi Normal University, Guilin, China; 3 Office of Development Planning, Guangxi College for Preschool Education, Nanning, China

**Keywords:** place attachment, nature connectedness, pro-environmental behavioral intentions, mediation model, questionnaire investigation, experimental manipulation

## Abstract

**Introduction:**

To investigate the influence of place attachment on pro-environmental behavioral intentions and the mediating role of nature connectedness.

**Methods:**

Study 1 employed a questionnaire survey method with 1,019 university students as research participants. Study 2 recruited 160 participants and used an experimental method to manipulate place attachment to examine its effects on nature connectedness and pro-environmental behavioral intentions.

**Results:**

The results of both studies consistently demonstrated that place attachment positively predicts pro-environmental behavioral intentions and nature connectedness partially mediates the relationship between place attachment and pro-environmental behavioral intentions. Specifically, place attachment not only directly promotes pro-environmental behavioral intentions but also indirectly facilitates such intentions through nature connectedness.

**Discussion:**

These findings indicate that fostering place attachment and a sense of connectedness to nature may serve as effective pathways for encouraging individuals to engage in more pro-environmental behaviors. This provides empirical evidence to inform the design of relevant interventions and the formulation of environmental policies.

## 1 Introduction

Due to the threats posed by global warming and the increasing frequency of extreme climate-related disasters, the Earth’s ecological environment is deteriorating at an unprecedented scale and pace. This process has directly or indirectly caused hundreds of species to face the risk of extinction, as well as economic damages exceeding billions of dollars (IPCC, 2021). As environmental degradation accelerates, although people have gradually become aware of the environmental crisis, but most individuals still do not engage in pro-environmental behaviors (Finger, 1994; Halpenny, 2010). Therefore, changing human behavioral patterns is essential for mitigating environmental deterioration (Poškus and Žukauskiene, 2017). Identifying ways to encourage individuals to adopt more pro-environmental behaviors has become a critical research and societal priority. While previous research has established a positive relationship between place attachment and pro-environmental behavior (Daryanto and Song, 2021; Scannell and Gifford, 2010a,b), several important gaps remain. First, most studies have focused on direct relationships with limited attention to underlying psychological mechanisms (Ramkissoon et al., 2013). Second, existing evidence is predominantly derived from North American countries characterized by individualistic cultural contexts (Ariccio et al., 2021; Halpenny, 2010), whereas validation within collectivistic cultures remains limited. Third, experimental evidence for causal relationships remains scarce, as most studies rely on correlational designs. Place attachment refers to the bond formed between individuals and important places (Lewicka, 2011; Scannell and Gifford, 2017a). According to interdependence theory (Kelley and Thibaut, 1978; Davis et al., 2009), people who develop attachment to specific places are personally affected when these places are harmed. The interdependent person-place relationship may motivate individuals to adopt more pro-environmental behaviors to protect the places to which they are attached (Scannell and Gifford, 2010b).

Furthermore, the internal mechanisms through which place attachment influences pro-environmental behavioral intentions have not been sufficiently discussed. Previous scholars have separately explored this from cognitive (Barbaro et al., 2015; Hernandez et al., 2010; Lee and Lee, 2017; Valizadeh et al., 2020) and affective perspectives (Jiao et al., 2023; Wang and Wu, 2015), with the limited investigation into composite psychological variables. Nature connectedness reflects the close relationship between humans and nature (Kals et al., 1999), encompassing both the cognitive closeness to nature and emotional attachment to it (Li et al., 2018). Existing research indicates that nature connectedness is not only a psychological consequence triggered by place attachment (Scannell and Gifford, 2017b) but also viewed as a source of hope for improving human’s pro-environmental behavior (Scannell and Gifford, 2010b; Zhou et al., 2023). Based on a review of relevant literature, the present study aims to verify the influence of place attachment on pro-environmental behavioral intentions and explore nature connectedness as a psychological mechanism mediating the relationship between the two.

### 1.1 Place attachment and pro-environmental behavioral intentions

To establish the theoretical foundation for our hypotheses, we first examine the relationship between place attachment and pro-environmental behavioral intentions. According to place attachment theory (Bowlby, 1969; Lewicka, 2011; Tuan, 1977), individuals with higher levels of place attachment demonstrate long-term, positive emotional connections and a willingness to protect places. Researchers consider place attachment to be a powerful predictor of pro-environmental behavioral intentions (Scannell and Gifford, 2010b; Zhang et al., 2014; Zhou et al., 2023). Empirical studies have provided supportive evidence, with relevant research indicating that place attachment is significantly positively correlated with pro-environmental behavioral intentions (Ramkissoon et al., 2013), adolescents with higher degrees of place attachment are more inclined to exhibit pro-environmental behaviors (Vaske and Kobrin, 2001), and surveys of different populations have found positive predictive effects of place attachment on pro-environmental behavior, such as among tourists (Cheng and Wu, 2015) and residents (Song and Soopramanien, 2019; Walker and Ryan, 2008). Furthermore, laboratory research has found that after experimental manipulation to activate subjects’ place attachment, the pro-environmental behavioral intentions of the attachment group increased compared to the control group (Jiao et al., 2023). However, research on the influence of place attachment on pro-environmental behavioral intentions has primarily focused on individualistic cultural contexts, lacking evidence from collectivistic cultural backgrounds (Daryanto and Song, 2021). Therefore, the present study aims to replicate previous findings within a collectivistic cultural context in order to obtain relatively stable results. Based on interdependence theory and existing empirical evidence (Scannell and Gifford, 2010b; Zhang et al., 2014), we propose Hypothesis 1: place attachment is positively associated with pro-environmental behavioral intentions.

### 1.2 The mediating role of nature connectedness

According to the biophilia hypothesis (Wilson, 1984), individuals have an innate preference for and affiliation with natural environments. This preference can be approximately expressed as a preference for natural environments over built environments (Mangone et al., 2017) or as time spent in nature (Chen et al., 2018). For places to which people are attached, whether residential areas (Crompton, 2001; Meidenbauer et al., 2019) or tourist destinations (Fleischer, 2012), individuals tend to interact more with nature. Compared to indirect experiences, direct contact between humans and nature is a more effective pathway to promoting nature connectedness (Li et al., 2018). Relevant research indicates that place attachment is significantly positively correlated with nature connectedness (Wilkie and Trotter, 2022); laboratory studies have found that place attachment positively predicts the attractiveness of natural environments to subjects (Kaltenborn and Bjerke, 2002); a longitudinal study also showed that compared to urban or indoor environments, subjects reported the highest levels of place attachment to parks, beaches, and other natural environments (Korpela et al., 2009); evidence from cognitive neuroscience demonstrates that place attachment induces activity in individuals’ amygdala and medial prefrontal cortex (mPFC), which is highly correlated with positive cognitive perceptions and emotions related to places (Gatersleben et al., 2020).

Moreover, nature connectedness may not only be a psychological consequence of place attachment but also an antecedent variable that further triggers pro-environmental behavioral intentions. Nature connectedness is likely to promote pro-environmental behavioral intentions (Hinds and Sparks, 2008; Whitburn et al., 2020; Zhou et al., 2023). The ecological self-theory (Naess, 1985) suggests that when people recognize nature as part of themselves, they form an ecological view that equates harming nature with harming themselves, thereby motivating them to protect nature (Yang et al., 2017). Empirical research provides supporting evidence: people who have established close relationships with nature in the past and have more positive memories of such experiences are more committed to pro-environmental behaviors (Kaffashi et al., 2015; Nisbet et al., 2009); people living in green buildings (e.g., with higher air quality, more natural light, and larger areas of vegetation) more easily establish place attachment and are consequently more inclined to engage in pro-environmental behaviors (Cole et al., 2021). Based on the above, we propose Hypothesis 2 of the present study: nature connectedness mediates the relationship between place attachment and pro-environmental behavioral intentions.

## 2 Study 1

### 2.1 Participants

As part of this research, we adopted a survey-based methodological approach to investigate the associations among place attachment, nature connectedness, and pro-environmental behavior intentions. Data were collected offline at a university located in Guangxi Province, China. Throughout the data collection process, we adhered to the principle of voluntary participation and recruited respondents through random sampling on campus. After obtaining informed consent, participants completed the questionnaire via an online survey platform using their mobile phones. All data were collected within a single working day to minimize potential temporal confounds. Questionnaires were distributed to 1,091 students, and 1,019 valid responses were collected, yielding an effective response rate of 93.4%. Among the participants, were male (22.7%) and 788 were female (77.3%); 278 were from urban areas (27.3%), and 741 were from rural areas (72.7%). The average age of participants was 20.58 years (SD = 2.84).

### 2.2 Measures

#### 2.2.1 Place attachment scale

The place attachment scale from Williams and Vaske (2003) was used, with participants reporting their place attachment to their current city of residence. The scale consists of 12 items encompassing two dimensions: place identity (e.g., I feel “X” is a part of me), and place dependence (e.g., “X” is the best place for what I like to do), Where “X” refers to the place of residence of the research subject. The scale employs a 7-point Likert scale, with ratings ranging from 1 (strongly disagree) to 7 (strongly agree). Higher scores indicate stronger place attachment to the current city of residence. In the present study, the Cronbach’s α coefficient for the scale was 0.91, with the place identity dimension having a Cronbach’s α coefficient of 0.94 and the place dependence dimension having a Cronbach’s α coefficient of 0.94 (Supplementary Appendix).

#### 2.2.2 Nature connectedness scale

The Chinese version of the nature connectedness scale revised by Li and Wu (2016) was adopted (e.g., I often feel a sense of oneness with the natural world around me.). This scale was originally developed by Mayer and Frantz (2004) and consists of 14 items rated on a 5-point Likert scale, with scores ranging from 1 (strongly disagree) to 5 (strongly agree). Higher scores indicate higher levels of connectedness with nature. In the present study, the Cronbach’s α coefficient for this scale was 0.85 (Supplementary Appendix).

#### 2.2.3 Pro-environmental behavior scale

The pro-environmental behavior scale supplementarily revised by Liu and Wu (2013) was used. It comprises 12 items covering two dimensions: public domain (e.g., Publicly expressing support for environmental protection, such as speeches, writing papers, etc.) and private domain behaviors (e.g., When there is no one in the room, leave the room and turn off the lights or fan actively). A Likert 5-point scoring system was employed, with higher scores indicating stronger pro-environmental behavioral intentions. In the present study, the Cronbach’s α coefficient for this scale was 0.86 (Supplementary Appendix).

### 2.3 Results

We first conducted descriptive statistics and correlation analyses of the major variables using SPSS 26.0. Subsequently, we employed the PROCESS macro (version 4.2) developed by Hayes (2017) to examine the mediating effects. Specifically, Model 4 from Hayes’ standard templates was selected to test the proposed mediation model, in which place attachment was entered as the independent variable, nature connectedness as the mediator, and pro-environmental behavioral intention as the dependent variable.

#### 2.3.1 Common method bias test

The present study collected data using questionnaires, which may be affected by common method bias. To minimize common method bias as much as possible, Harman’s single-factor test was used for post-statistical control. The results showed that there were 5 factors with eigenvalues greater than 1 without rotation, and the maximum explanatory rate of the first common factor was 32.92%, which is less than 40%. This indicates that there was no serious common method bias problem in the present study.

#### 2.3.2 Descriptive statistics and correlation analysis

**FIGURE 1:**
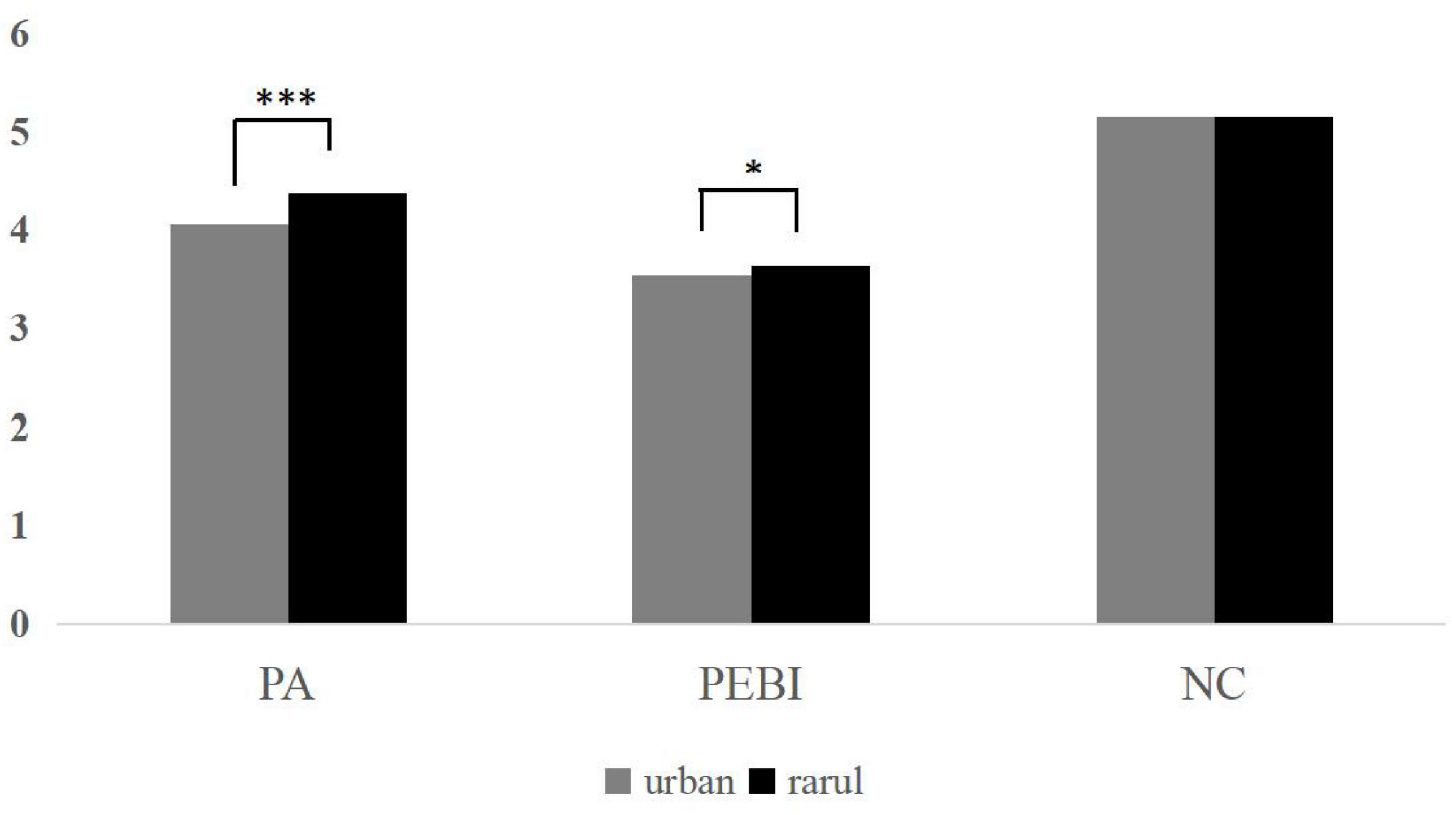
Distribution of main variables in urban/rural areas. PA, place attachment; NC, nature connectedness; PEBI, pro environmental behavioral intension. *p < 0.05, **p < 0.01, ***, *p* < 0.001.

**FIGURE 2:**
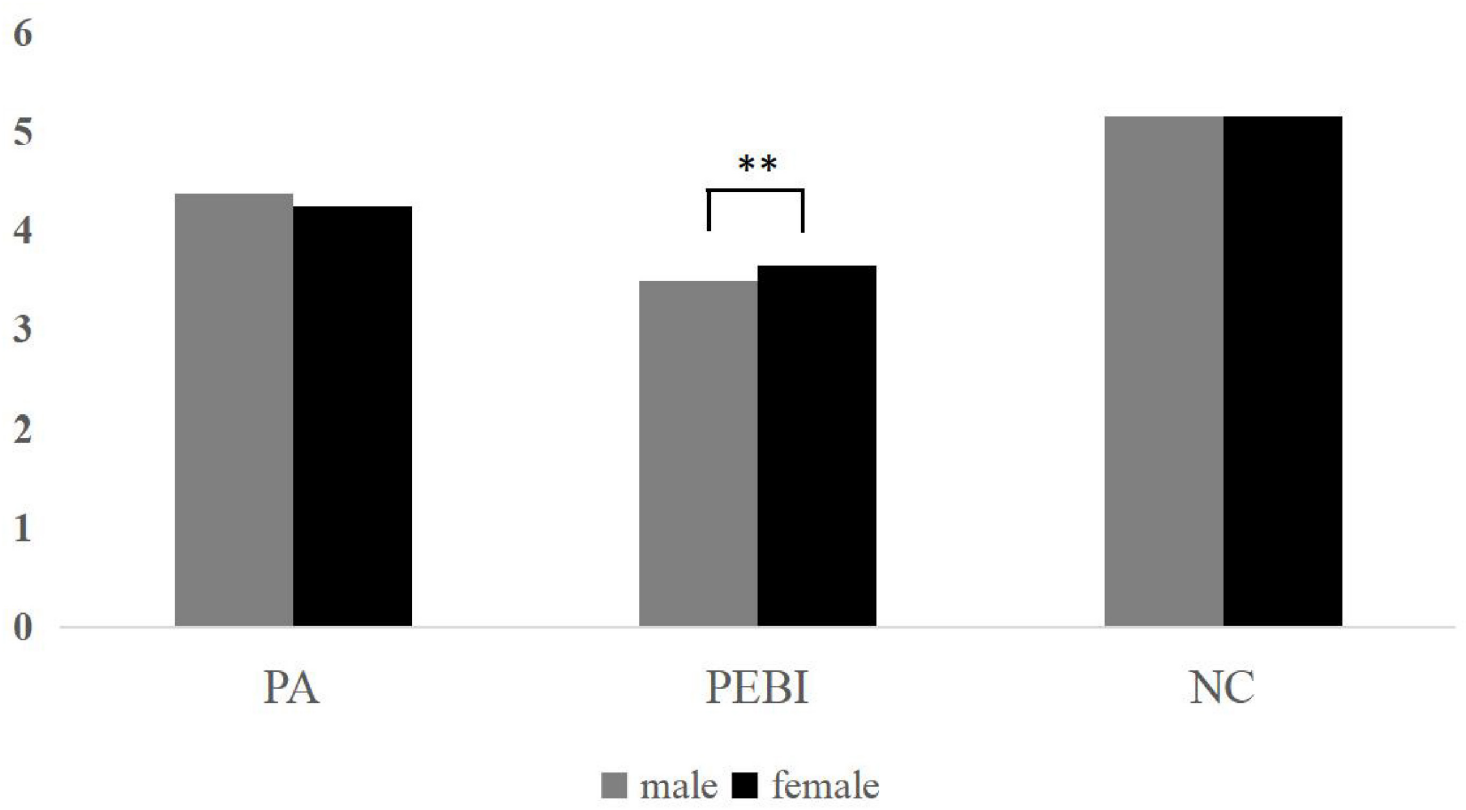
Distribution of main variables in male/female areas. PA, place attachment; NC, nature connectedness; PEBI, pro environmental behavioral intension. *p < 0.05, **p < 0.01, *** *p* < 0.001.

The distributions of major variables across demographic characteristics are illustrated in the Figures 1, 2. Participants from urban (*M* = 4.06) and rural areas (*M* = 4.36) differed significantly in place attachment (*p* < 0.001). Significant differences were also observed in PEBI between urban (*M* = 3.54) and rural participants (*M* = 3.64; *p* = 0.046), whereas nature connectedness did not differ significantly between the two groups. Furthermore, male (*M* = 3.49) and female (*M* = 3.64) participants differed significantly in PEBI (*p* = 0.005), while no significant gender differences were found for place attachment or nature connectedness.

The descriptive statistics and correlations among variables are shown in Table 1: place attachment was significantly positively correlated with nature connectedness (*r* = 0.33, *p* < 0.001) and pro-environmental behavioral intentions (*r* = 0.36, *p* < 0.001); nature connectedness was significantly positively correlated with pro-environmental behavioral intentions (*r* = 0.51, *p* < 0.001).

**TABLE 1:** Description of variables and correlation analysis.

Variables	*M*	SD	1	2	3	4	5
1. Age	20.58	2.84	–	–	–	–	
2. Gender	–	–	0.08*				
3. Residence	–	–	0.07*	0.03			
4. Place attachment	4.28	1.12	0.03	−0.07*	−0.12***		
5. Nature connectedness	5.16	0.89	0.03	0.08*	0.01	0.33***	–
6. PEBI	3.61	0.66	0.01	−0.09*	−0.06	0.36***	0.51***

*p < 0.05,

***p < 0.001, PEBI, pro-environmental behavioral intentions.

#### 2.3.3 Mediating effect of nature connectedness

The PROCESS macro in SPSS using Model 4 was employed for mediating effect analysis, controlling for age, gender, and family origin. With place attachment as the independent variable and pro-environmental behavioral intentions as the dependent variable, after introducing nature connectedness into the model, the results shown in Table 2 indicate that place attachment significantly predicted pro-environmental behavioral intentions (95% CI = [0.08, 0.19]) and nature connectedness (95% CI = [0.38, 0.48]), and nature connectedness positively predicted pro-environmental behavioral intentions (95% CI = [0.48, 0.60]).

**TABLE 2:** Mediation analysis in Study 1.

Predictive variables	Nature connectedness			Pro-environmental behavioral intentions		
	β	*t*	95% CI	β	*t*	95% CI
Place attachment	0.43	17.11***	[0.38, 0.48]	0.13	4.55***	[0.08, 0.19]
Nature connectedness				0.54	16.99***	[0.48, 0.60]
*R* ^2^	0.22			0.34		
*F*	73.30***			102.46***		

***p < 0.001.

The bootstrap 95% confidence intervals for both the direct effect of place attachment on pro-environmental behavioral intentions and the mediating effect of nature connectedness did not contain 0, as shown in Table 3. The direct effect (0.23) accounted for 64% of the total effect (0.36), while the mediating effect (0.13) accounted for 36% of the total effect (0.36).

**TABLE 3:** Decomposition of total effect, direct effect, and mediating effect.

Effect type	Effect value	Standard error	Lower limit	Upper limit	Relative effect value
Total effect	0.36	0.03	0.31	0.42	100%
Direct effect	0.23	0.03	0.08	0.19	64%
Mediating effect	0.13	0.02	0.20	0.27	36%

These results indicate that place attachment not only has a direct predictive effect on individuals’ pro-environmental behavioral intentions but also indirectly predicts pro-environmental behavioral intentions through nature connectedness, providing support for Hypothesis 1 and Hypothesis 2.

### 2.4 Summary

Study 1 used a questionnaire method to confirm the correlation between place attachment and pro-environmental behavioral intentions, as well as the mediating role of nature connectedness. However, correlational research makes it difficult to infer causal relationships among variables (Rottman and Hastie, 2014). Therefore, to address these limitations to some extent and better reveal the causal relationships of place attachment’s influence on pro-environmental behavioral intentions and its internal mechanisms, Study 2 will employ an experimental method to manipulate participants’ place attachment.

## 3 Study 2

Study 2 explored whether place attachment has the same predictive effect on pro-environmental behavioral intentions and whether nature connectedness mediates the relationship between place attachment and pro-environmental behavioral intentions by experimentally manipulating participants’ place attachment. The present study expected that participants in the attachment condition (compared to the control group) would report higher levels of pro-environmental behavioral intentions. While Study 2 employed a different pro-environmental behavior scale [adapted from Tonge et al. (2015)] compared to Study 1 (Liu and Wu, 2013), both scales assess behavioral intentions rather than actual behaviors and cover similar domains of environmental action. This approach allows us to examine the robustness of our findings across different operationalizations of the construct.

### 3.1 Participants

G*Power 3.1 software was used to calculate the required sample size for the experiment. At a significance level of α = 0.05 and with a medium effect size, a total sample size of at least 146 participants was needed to achieve a statistical power of 0.85. A total of 160 participants were recruited from a university in Guangxi, including 68 males and 92 females, who were randomly assigned to groups. The mean age of participants was 23.68 years (SD = 8.89). Among them, 80 were assigned to the place attachment group and 80 to the control group. Prior to the manipulation, random assignment ensured that participants in the place attachment and non-attachment conditions were initially equivalent in key demographic variables and baseline environmental attitudes, supporting the internal validity of the comparison.

### 3.2 Experimental design and procedure

The present study employed a single-factor, between-subjects experimental design. Place attachment (attachment vs. control) served as the independent variable, nature connectedness as the mediator, and pro-environmental behavioral intention as the dependent variable. Consistent with previous research, we adopted an adapted imagery paradigm combined with a written reflection task to manipulate participants’ levels of place attachment (Ariccio et al., 2021; Jiao et al., 2023; Scannell and Gifford, 2017b). To ensure procedural clarity and replicability, all participants received standardized instructions prior to completing the imagine task. They were informed that they would spend 5 min imagining and writing about a specific place based on the provided guidelines. In the attachment condition, participants were instructed to recall a place that held strong positive emotional significance and to describe (1) the name of the place, (2) its meaningful features or characteristics, and (3) the positive psychological experiences associated with being in that place (minimum 20 words). One representative response noted:

“*My hometown county, where I spent most of my childhood holidays. It also holds memories of my 6 years of secondary school. Every time I return, I feel a profound sense of stability; it is the place where I truly feel I belong*.”

In contrast, participants assigned to the control condition were asked to imagine and write only the name or location of an ordinary, emotionally neutral place. A representative response stated: “*An ordinary road near my home. It is a busy city street with heavy traffic and many people. Tall buildings and loud crowds are everywhere.*”

#### 3.2.1 Experimental manipulation and verification materials

In the present study, different instructions were used for the attachment condition (e.g., “In our lives, there are some places that are distinct from other physical spaces and have specific meaning to us. Please imagine a place that has positive meaning and emotional connection for you, …”) and the non-attachment condition (e.g., “In our lives, there are some places that are distinct from other physical spaces and have specific meaning to us. Another part of places are just ordinary geographical spaces. Please randomly imagine a place that is very ordinary to you, …”). Following the imagine task, participants completed four manipulation-check items (e.g., “At this moment, I feel a strong sense of identification with this place”), demonstrating acceptable internal consistency (Cronbach’s α = 0.80). Participants then completed the nature connectedness scale and the pro-environmental behavioral intention scale, followed by demographic questions regarding gender, age, and place of residence.

#### 3.2.2 Nature connectedness measurement

Nature connectedness was measured using the same scale as in Study 1. The scale assesses individuals’ subjective sense of connection, belonging, and emotional closeness to the natural world (e.g., I often feel a sense of oneness with the natural world around me). Participants rated items on a 7-point Likert scale (1 = strongly disagree, 7 = strongly agree). Higher scores indicated higher perceived connectedness with nature. In the present study, Cronbach’s α for this scale was 0.80, indicating good internal consistency.

#### 3.2.3 Pro-environmental behavioral intentions measurement

In the present study, the pro-environmental behavior scale was adapted from previous research (Tonge et al., 2015) to assess participants’ pro-environmental intentions. It comprised a total of 9 items across two dimensions. Four items were used to measure the behaviors that the subjects themselves would do (e.g., Learn more about this place natural environment), Five items measured subjects’ intention to engage in protective behavior (e.g., Work as a volunteer on conservation projects in this area), A 5-point scoring system was used (1 = strongly disagree, 5 = strongly agree), with higher scores indicating stronger pro-environmental behavioral intentions. In the present study, Cronbach’s α for this scale was 0.86.

### 3.3 Results

#### 3.3.1 Manipulation check for place attachment and main effect test

Participants in the place attachment group were coded as 1, and those in the control group were coded as 0. Independent samples *t*-test was used to examine the effectiveness of the place attachment manipulation. The results shown in the table below indicate that the place attachment reported by the experimental group (3.93 ± 0.59) was significantly higher than that of the control group (2.99 ± 0.86), (*t*(160) = 7.96, *p* < 0.001, Cohen’s *d* = 1.27, 95% CI [0.79, 1.31]), suggesting that the manipulation of place attachment was effective.

Participants in the attachment condition (vs. control group) reported higher levels of nature connectedness (*t*(160) = 2.59, *p* < 0.05, Cohen’s *d* = 0.41, 95% CI = [0.10, 0.71]) and higher pro-environmental behavioral intentions (*t*(160) = 2.05, *p* < 0.05, Cohen’s *d* = 0.33, 95% CI = [0.02, 0.62]).

#### 3.3.2 Mediation effect test

**FIGURE 3:**
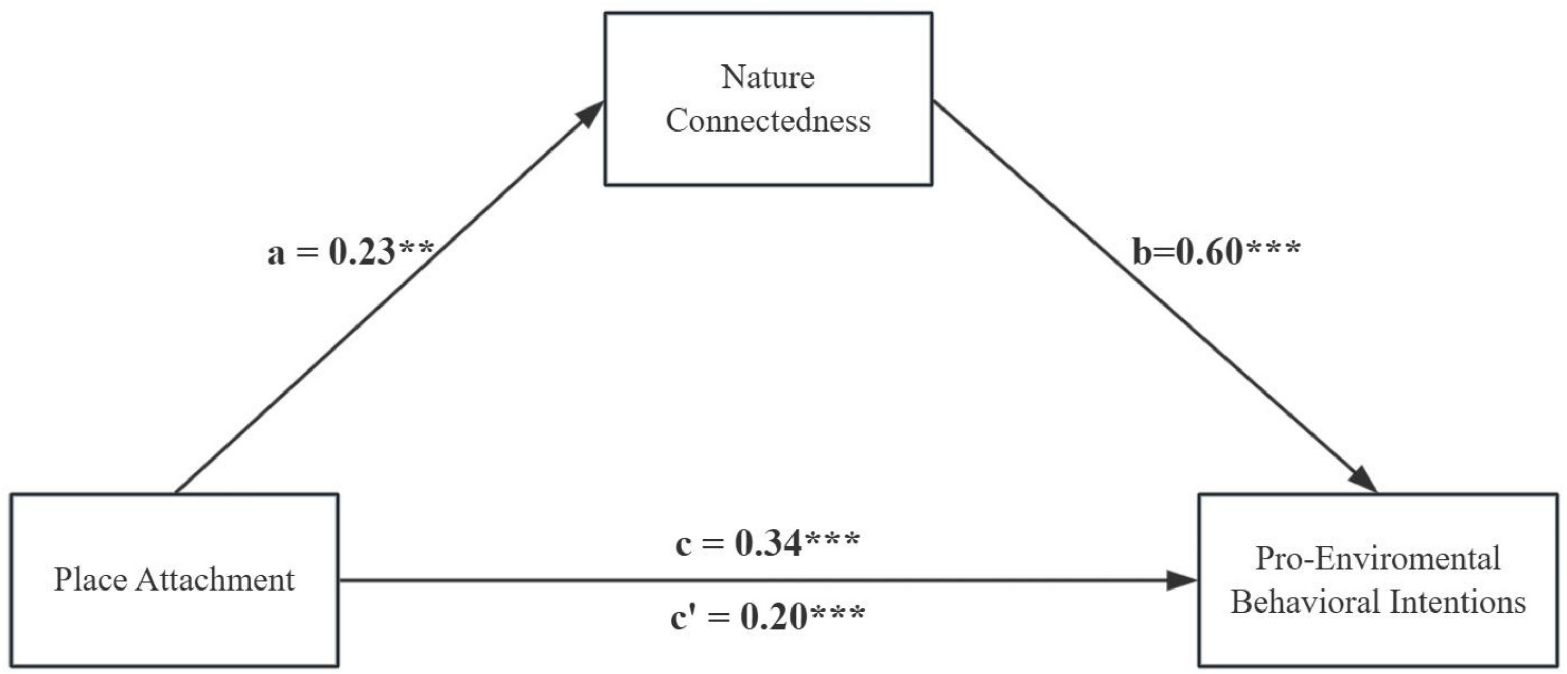
The mediation effect of nature connectedness. **p < 0.01, ***p < 0.001.

SPSS 26.0 software and the PROCESS v4.2 macro program developed by Hayes (2017) were used for data analysis, with Model 4 from Hayes’ typical models selected to test the mediating role of nature connectedness in the relationship between place attachment and pro-environmental behavioral intentions. The results (see Figure 3) showed that the direct effect of place attachment on pro-environmental behavioral intentions was significant (β = 0.23, *t* = 3.40, 95% CI = [0.08, 0.39]), and the mediating effect of nature connectedness between place attachment and pro-environmental behavioral intentions was also significant (β = 0.20, 95% CI = [0.09, 0.32]). The direct effect (0.20) and the mediating effect (0.14) accounted for 58.82% and 41.18% of the total effect (0.41), respectively. This indicates that nature connectedness mediated the relationship between place attachment and pro-environmental behavioral intentions.

### 3.4 Summary

Study 2 manipulated place attachment using an adapted imagination paradigm and writing task, providing stronger causal evidence for the relationships among place attachment, nature connectedness, and pro-environmental behavioral intentions. The results revealed that participants in the place attachment condition self-reported more pro-environmental behavioral intentions than those in the non-attachment condition, indicating that place attachment has a positive influence on pro-environmental behavioral intentions, and nature connectedness plays a partial mediating role in this relationship.

## 4 General discussion

### 4.1 The impact of place attachment on pro-environmental behavioral intentions

The present study investigated whether and how place attachment influences individuals’ pro-environmental behavioral intentions. Previous studies have demonstrated that place attachment can function as a stable trait (Williams and Vaske, 2003), and it can also be experimentally manipulated as state place attachment (Jiao et al., 2023). Through two progressive studies, the present research measured participants’ trait place attachment (Study 1) and manipulated state place attachment (Study 2), yielding relatively robust and consistent results. Study 1 measured place attachment as a disposition (i.e., place identity and place dependence), Study 2 examined state place attachment (“in this moment”). This distinction has theoretical implications: trait measures capture individuals’ stable bonds with meaningful places, whereas state induction reflects momentary feelings of connection elicited by situational cues. The findings of Study 2 demonstrate that even short-term, situationally induced place attachment can enhance pro-environmental behavioral intentions, and nature connectedness partially mediates this effect. This addresses, to some extent, the call by earlier scholars for empirical research on the impact of increased place attachment on pro-environmental behavioral intentions (Scannell and Gifford, 2017a) and validates previous findings in a collectivistic cultural context. This finding is consistent with previous research results (Jiao et al., 2023) and earlier place attachment theory (Relph, 1976), which suggests that the attachment people establish to places motivates them to develop intentions to protect these places. According to the tripartite framework of place attachment (Scannell and Gifford, 2010a), the three dimensions of this framework include: (1) the person/culture dimension, with the present study focusing on place attachment among Chinese participants in a collectivistic cultural background. In collectivistic cultures such as China, place attachment may be strengthened through shared family histories and community ties that span generations (Hwang, 2001). Unlike individualistic cultures where place attachment often reflects personal identity and autonomy, collectivistic place attachment may be more intertwined with family obligations and community harmony, potentially creating stronger motivations for environmental protection of shared spaces; (2) the place dimension, where “place” not only has a social dimension but is also a tangible, real physical environment (Kaltenborn, 1997). Previous research linking people’s emotional bonds to the physical attributes of place has been very scarce (Lewicka, 2011). The present study explicitly incorporates the physical environmental characteristics of places–specifically, natural features–into the examination of person–place relationships. Although the experimental paradigm was primarily designed to manipulate place attachment rather than to measure physical characteristics directly, participants’ imagination-task responses (Study 2) revealed that natural elements were frequently and spontaneously referenced in descriptions of meaningful places. For instance, participants in the attachment condition commonly described natural attributes such as trees, riversides, lakeside paths, mountains, seasonal vegetation, and specific natural sensory qualities (e.g., breeze, sunlight, sounds of water or leaves). These naturally emerging descriptions suggest that, for many individuals, significant places are closely intertwined with natural environmental features; (3) the psychological process dimension, including behavioral motivations to protect places, love for nature, and specific types of behaviors (e.g., pro-environmental behaviors), which to some extent supplements and enriches the literature in related fields.

### 4.2 The mediating role of nature connectedness

The results of both studies consistently demonstrate that nature connectedness partially mediates the relationship between place attachment and pro-environmental behavioral intentions. Attention Restoration Theory (Kaplan, 1995) suggests that natural environments facilitate the restoration of psychological resources, and these positive emotional experiences may be triggered by individuals’ emotional bonds with specific places (Scannell and Gifford, 2017a; Yoshida et al., 2022), thereby contributing to understanding individuals’ preferences for interacting with nature and why they make pro-environmental behavioral choices (Zhou et al., 2023). Research indicates that place attachment is an antecedent variable for perceiving the restorativeness of natural environments (Liu et al., 2020).

Recent laboratory studies have also found that natural environments provide numerous benefits to people, such as enhancing authenticity (Yang et al., 2024), reducing stress (Korpilo et al., 2024), improving mood (Coventry et al., 2021), and promoting psychological health and well-being (Yang et al., 2022). These findings help us understand the altruistic mechanisms, such as pro-environmental behaviors, triggered by nature. The mediation of nature connectedness in the effect of place attachment on pro-environmental behavioral intentions suggests that in daily life, we should guide people to establish emotional attachments to their locations by, for example, improving the physical environment of places, constructing place landmarks, collective meanings, and spatial memories (Stedman, 2002), thereby increasing people’s emotional connection to places and promoting individuals’ pro-environmental behavioral intentions. We can also enhance people’s nature connectedness through increasing green landscapes and incorporating more environmentally friendly designs, which is conducive to improving pro-environmental behavioral intentions in the context of place attachment.

## 5 Limitations and future directions

Our study provides empirical support for the promotion of pro-environmental behavioral intentions by place attachment and its internal mechanisms through two progressive studies, offering certain insights while acknowledging limitations. People’s levels of place attachment may change over time (Li and McKercher, 2016). Future research could attempt to adopt longitudinal research methods to explore the long-term effects of place attachment on pro-environmental behavior. In addition, the present study measured pro-environmental behavioral intentions through self-report measures, which may be subject to unavoidable social desirability bias (Kormos and Gifford, 2014). Previous scholars have pointed out that behavioral intentions do not always shape individual behavior (Li et al., 2019).

Future research could consider using computer experiments (PEBT) or field observations to measure pro-environmental behavior (Lange et al., 2018; Lange and Dewitte, 2019). Finally, the samples selected for the present study were all Chinese participants with collectivistic cultural backgrounds. Caution should be exercised when generalizing the conclusions of The present study to other samples or cultural contexts. Future research could consider conducting cross-cultural studies.

## Data Availability

The original contributions presented in this study are included in this article/Supplementary material, further inquiries can be directed to the corresponding author.
