# Supplementary material for: The relationship between place attachment and pro-environmental behavioral intentions: the mediating role of nature connectedness
Source: Front Psychol. 2026 Jan 12;16:1678177. doi: 10.3389/fpsyg.2025.1678177 (PMC12832409; doi:10.3389/fpsyg.2025.1678177)
Supplement: Supplementary file 1 [file Table_1.docx]

**Appendix**

**Study 1**

**Place attachment Scale (“X” refers to the specific location)**

| items | Strongly disagree | Disagree | Somewhat disagree | Neutral | Somewhat agree | agree | Strongly agree |
| --- | --- | --- | --- | --- | --- | --- | --- |
| 1. I feel “X” is a part of me. | **1** | **2** | **3** | **4** | **5** | **6** | **7** |
| 2. “X” is very special to me. | **1** | **2** | **3** | **4** | **5** | **6** | **7** |
| 3. I identify strongly with “X”. | **1** | **2** | **3** | **4** | **5** | **6** | **7** |
| 4. I am very attached to “X”. | **1** | **2** | **3** | **4** | **5** | **6** | **7** |
| 5. Visiting “X” says a lot about who I am. | **1** | **2** | **3** | **4** | **5** | **6** | **7** |
| 6. “X” means a lot to me. | **1** | **2** | **3** | **4** | **5** | **6** | **7** |
| 7. “X” is the best place for what I like to do. | **1** | **2** | **3** | **4** | **5** | **6** | **7** |
| 8. No other place can compare to “X”. | **1** | **2** | **3** | **4** | **5** | **6** | **7** |
| 9. I get more satisfaction out of visiting “X” than any other. | **1** | **2** | **3** | **4** | **5** | **6** | **7** |
| 10. Doing what I do at “X” is more important to me than doing it in any other place. | **1** | **2** | **3** | **4** | **5** | **6** | **7** |
| 11. I wouldn't substitute any other area for doing the types of things I do at “X”. | **1** | **2** | **3** | **4** | **5** | **6** | **7** |
| 12. The things I do at “X” I would enjoy doing just as much at a similar site. | **1** | **2** | **3** | **4** | **5** | **6** | **7** |

**Nature connectedness Scale**

| items | Strongly disagree | Disagree | Somewhat disagree | Neutral | Somewhat agree | agree | Strongly agree |
| --- | --- | --- | --- | --- | --- | --- | --- |
| **1.**I often feel a sense of oneness with the natural world around me. | **1** | **2** | **3** | **4** | **5** | **6** | **7** |
| **2.**I think of the natural world as a community to which I belong. | **1** | **2** | **3** | **4** | **5** | **6** | **7** |
| **3.**I recognize and appreciate the intelligence of other living organisms | **1** | **2** | **3** | **4** | **5** | **6** | **7** |
| **4.** I often feel disconnected from nature. | **1** | **2** | **3** | **4** | **5** | **6** | **7** |
| **5.** When I think of my life, I imagine myself to be part of a larger cyclical process of living. | **1** | **2** | **3** | **4** | **5** | **6** | **7** |
| **6.** I often feel a kinship with animals and plants. | **1** | **2** | **3** | **4** | **5** | **6** | **7** |
| **7.** I feel as though I belong to the Earth as equally as it belongs to me. | **1** | **2** | **3** | **4** | **5** | **6** | **7** |
| **8.** I have a deep understanding of how my actions affect the natural world. | **1** | **2** | **3** | **4** | **5** | **6** | **7** |
| **9.** I often feel part of the web of life. | **1** | **2** | **3** | **4** | **5** | **6** | **7** |
| **10.** I feel that all inhabitants of Earth, human, and nonhuman, share a common ‘life force’. | **1** | **2** | **3** | **4** | **5** | **6** | **7** |
| **11.** Like a tree can be part of a forest, I feel embedded within the broader natural world. | **1** | **2** | **3** | **4** | **5** | **6** | **7** |
| **12.** When I think of my place on Earth, I consider myself to be a top member of a hierarchy that exists in nature. | **1** | **2** | **3** | **4** | **5** | **6** | **7** |
| **13.** I often feel like I am only a small part of the natural world around me, and that I am no more important than the grass on the ground or the birds in the trees. | **1** | **2** | **3** | **4** | **5** | **6** | **7** |
| **14.** My personal welfare is independent of the welfare of the natural world. | **1** | **2** | **3** | **4** | **5** | **6** | **7** |

**Pro environmental Behavior Intention Scale**

| items | Never | Rarely | Occasionally | Sometimes | Often |
| --- | --- | --- | --- | --- | --- |
| 1. Publicly expressing support for environmental protection (such as speeches, writing papers, etc.) | **1** | **2** | **3** | **4** | **5** |
| 2. Discussing environmental issues with others. | **1** | **2** | **3** | **4** | **5** |
| 3. Reuse of plastic bags. | **1** | **2** | **3** | **4** | **5** |
| 4. Actively participate in activities organized by schools or environmental protection societies. | **1** | **2** | **3** | **4** | **5** |
| 5. Bring your own shopping bag when buying daily necessities. | **1** | **2** | **3** | **4** | **5** |
| 6. Actively participate in various forms of environmental protection publicity and education activities. | **1** | **2** | **3** | **4** | **5** |
| 7. When there is no one in the room, leave the room and turn off the lights or fan actively | **1** | **2** | **3** | **4** | **5** |
| 8. Save empty drink bottles, wine bottles, etc. and sell them | **1** | **2** | **3** | **4** | **5** |
| 9. Save empty drink bottles, wine bottles, etc. and sell them | **1** | **2** | **3** | **4** | **5** |
| 10. Advise others to stop destroying the environment (such as littering, sewage discharge, etc.). | **1** | **2** | **3** | **4** | **5** |
| 11. Use waste paper and the other side of the printing paper again | **1** | **2** | **3** | **4** | **5** |
| 12. Try not to use disposable personal items | **1** | **2** | **3** | **4** | **5** |

**Study 2**

**Manipulation Check**

| items | Strongly disagree | Disagree | Neutral | Agree | Strongly agree |
| --- | --- | --- | --- | --- | --- |
| 1. I identify strongly with this place. | **1** | **2** | **3** | **4** | **5** |
| 2. I am very attached to this place to measure place identity. | **1** | **2** | **3** | **4** | **5** |
| 3. Doing what I do at this place is more important to me than doing it at any other place | **1** | **2** | **3** | **4** | **5** |
| 4. The things I do at this place I would enjoy doing just as much at a similar place. | **1** | **2** | **3** | **4** | **5** |

**Pro environmental Behavior Intention Investigation**

| items | Strongly disagree | Disagree | Neutral | Agree | Strongly agree |
| --- | --- | --- | --- | --- | --- |
| 1. Learn more about this place natural environment. | **1** | **2** | **3** | **4** | **5** |
| 2. Consciously conserve water in my daily activities. | **1** | **2** | **3** | **4** | **5** |
| 3. Restrict my vehicle movements to designated access tracks. | **1** | **2** | **3** | **4** | **5** |
| 4. Place my cans and glass bottles in campsite recycling bins (if provided) | **1** | **2** | **3** | **4** | **5** |
| 5. Work as a volunteer on conservation projects in this area. | **1** | **2** | **3** | **4** | **5** |
| 6. Sign petitions in support of the conservation on place. | **1** | **2** | **3** | **4** | **5** |
| 7. Donate money to conservation projects to help protect this place. | **1** | **2** | **3** | **4** | **5** |
| 8. Write letters in support of the conservation of this place. | **1** | **2** | **3** | **4** | **5** |
| 9. Circulate petitions in support of the conservation of this place. | **1** | **2** | **3** | **4** | **5** |
